# Blast Resistance of 240 mm Building Wall Coated with Polyurea Elastomer

**DOI:** 10.3390/ma15030850

**Published:** 2022-01-23

**Authors:** Long Ji, Ping Wang, Youer Cai, Wei Shang, Xudong Zu

**Affiliations:** 1 Xi’an Modern Control Technology Research Institute, Xi’an 710065, China; jilong1683@hotmail.com (L.J.); zdb20311@163.com (P.W.); 2 School of Mechanical Engineering, Nanjing University of Science and Technology, Nanjing 210094, China; caiyouer@njust.edu.cn (Y.C.); ammosw0257@njust.edu.cn (W.S.)

**Keywords:** polyurea elastomer, brick wall, explosive load, simulation

## Abstract

Enhancing the blast resistance of building walls is a research hotspot in the field of anti-terrorism and explosion protection. In this study, numerical simulation and experimental verification were combined to analyze the failure phenomenon of brick masonry wall and sprayed polyurea-reinforced brick wall under contact explosion and determine the failure response parameters of the wall. The failure limit, mode, and mechanism of a 240 mm wall without reinforcement and strengthened with polyurea elastomer under different strength loads were investigated. Under contact explosion, the increase in the size of the blasting pit of the 240 mm wall gradually slowed down after the dose was increased to higher than 0.5 kg. Thereafter, the energy of the explosive load was released by splashing wall fragments as well as by deflecting and movement of the wall. The results show that the 240 mm walls sprayed with polyurea elastomer had outstanding anti-explosion performance because it wraps the damaged area and fragments of masonry wall inside the polyurea layer. When the thickness of the polyurea layer increases to 8 mm, the damaged area of the masonry wall decreases by 55.6% compared with that without reinforcement. The numerical simulation results were in good agreement with the experimental results.

## 1. Introduction

In today’s world, terrorist incidents frequently occur, and terrorist attacks on buildings have never stopped. The walls of industrial and civil buildings are mostly made of clay bricks. which is a typical brittle material and cannot absorb a lot of energy to slow down the damage of shock wave. The explosive load will cause damage to the wall, and a large avalanche will form on the back of the wall, which will seriously threaten the lives of people in the buildings. Therefore, research on enhancing the explosion-proof capability of the wall without changing the basic structure of existing buildings is important. Polymer coating can enhance the explosion-proof performance of an existing wall structure, which has made the explosive shock response characteristics of the sprayed polymer wall become a research hotspot [1]. Polyurea has excellent impact resistance and tear resistance and has great application potential in the field of anti-terrorism and explosion protection. Polyurea sprayed on the surface of the wall can absorb the energy of the explosion shock wave and reduce the secondary damage caused by the wall collapse during the explosion [2,3].

To reduce the damage to the wall by the explosion, scholars from various countries have conducted research on the blast-resistant performance of the wall. Kai X et al. simulated the explosive impact response of reinforced concrete walls and confirmed that the back reinforcement of concrete walls can effectively reduce wall caving [4]. Lee G. Moradi et al. studied the response characteristics of a masonry wall when subjected to lateral force impact [5]. Milani G et al. presented a simple rigid-plastic homogenization model for the analysis of masonry structures subjected to out-of-plane impact loads [6]. Silva L.C. et al. use a simple and reliable Homogenization approach coupled with a HRBSM accounting for high strain rate effects to analyze masonry panels subjected to impact and blast loads [7]. Sanam Aghdamy et al. studied the enhancement effect of polymers on the performance of masonry wall by simulation and concluded that the polymers can effectively improve the resistance of the masonry wall to an explosion load [8]. James S. Davidson et al. determined the damage mechanism and conducted experimental research of the coated polymer masonry wall [9,10]. The results show that the coated polymers wall can significantly enhance the explosion-proof performance of the masonry wall. 

However, there studies on the anti-explosion performance of wall with different coating thickness of polyurea elastomer are still in small numbers. In this work, the damage and failure characteristics of different polyurea coating methods to strengthen different walls under different intensities of contact explosion loads were studied through numerical simulation and experiment. The selected chemical amount would be greater than or equal to the critical penetration value of the bare masonry wall to compare and verify the blast-resistant effect of the polyurea elastomer coating and ensure that the corresponding unreinforced masonry wall is penetrated. Contact explosion test verification was carried out on the ordinary clay masonry wall reinforced by spraying polyurea under unidirectional support conditions. Two TNT explosives of different qualities were selected for the test, and the results were compared with the numerical simulation to analyze the working mechanism and response characteristics. The structural effects of the coating method were also analyzed to obtain a coating method for spraying polyurea reinforced masonry wall with excellent blast-resistant performance.

## 2. Numerical Simulation of Blast-Resistant Performance of 240 mm Wall under Contact Explosion

### 2.1. Numerical Simulation Model

**Figure 1:**
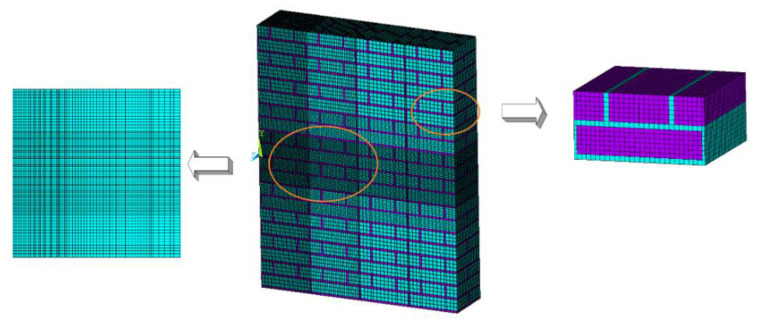
1/2 Model and mesh generation of 240 mm wall.

A separate model was used to describe the 240 mm masonry wall. Bricks and mortar were made of different material units and regarded as different parts in the solution. Simulation was conducted in accordance with the masonry regulations of masonry wall in relevant standards. The standard brick size used was: 240 mm × 115 mm × 53 mm. The repeated unit model consists of four bricks and adjacent mortar; the size was 240 mm × 250 mm × 126 mm as shown in the right part of Figure 1. The size of the single masonry wall was 1990 mm × 1260 mm × 240 mm, and the size of 1/2 model of masonry wall was 995 mm × 1260 mm × 240 mm. The explosive was a TNT cylindrical explosive with a charge density of 1.63 g/cm^3^. 

(1) Explosive and air material model and parameters

The three-dimensional structural calculation model constructed the Euler grid of explosives and air and the Lagrange grid of the wall structure and used the Euler/Lagrange full contact algorithm to simulate the impact of the shock wave and the structure. In the numerical simulation calculation, air adopted the ideal gas state model, and explosives were expressed by the JWL equation of state as follows [7]:

$$P=A\left(1-\frac{\omega }{R_{1}V}\right)e^{-R_{1}V}+B\left(1-\frac{\omega }{R_{2}V}\right)e^{-R_{2}V}+\frac{\omega E}{V}$$

where *P* is the pressure of the explosive detonation products, *E* is the internal energy per unit mass of the explosive, *V* is the relative volume, and *A*, *B*, *R*_1_, *R*_2_, and *ω* are the material parameters of the explosive. The diameter of the TNT explosive charge column is 100 mm, and the material parameters and state equation settings are shown in Table 1 and Table 2.

(2) Material model and parameters of the masonry wall

The masonry wall material used the MAT_ADD_EROSION option to simulate the failure of brickwork and mortar. The principal strain criterion was used as the failure criterion for brickwork and mortar, and the material parameters and state equation settings of block and mortar are shown in Table 3.

### 2.2. Damage of the Wall at Different Times

**Figure 2:**
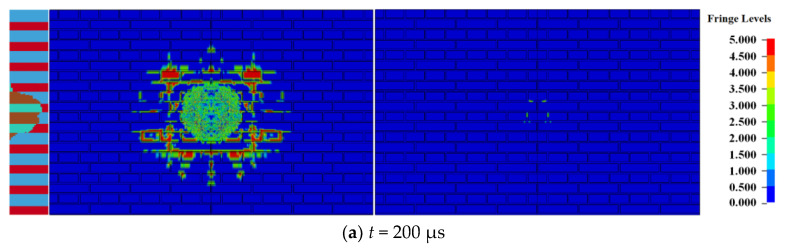

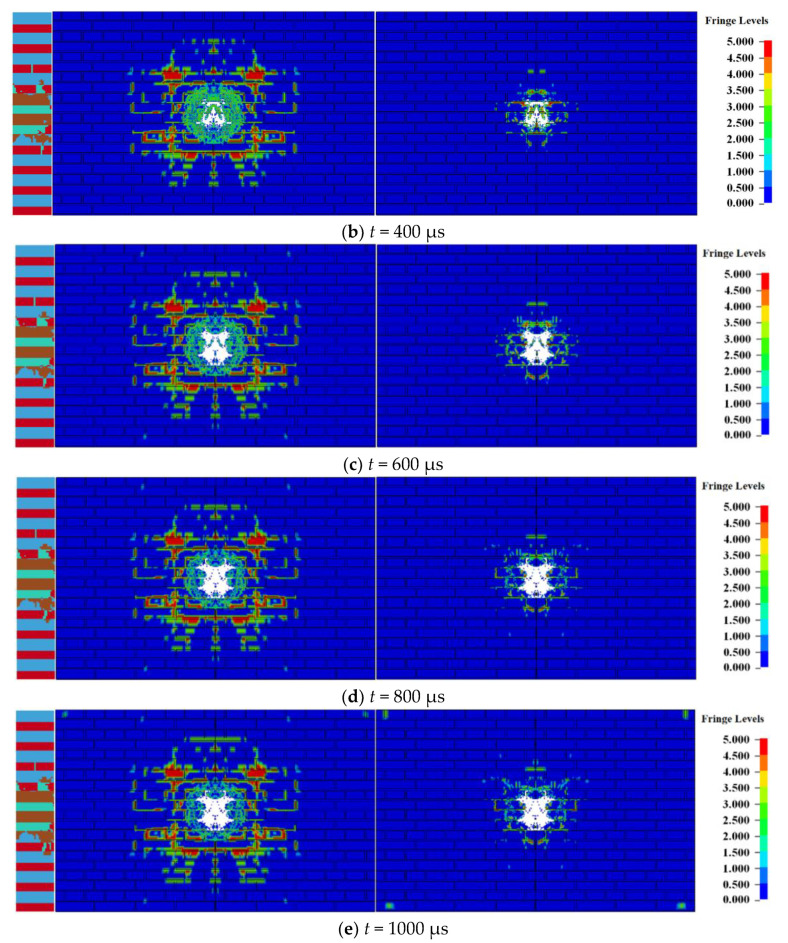
Damage of the 240 mm wall at different times.

When the amount of TNT was 0.5 kg, the damage of the 240 mm wall at different times is shown in Figure 2, where the left, middle, and right sides of the figure reflect the destruction of the longitudinal section (i.e., symmetrical plane), the blast front surface, and the blast back surface of the masonry wall.

After contact explosion between the explosive and the masonry wall, due to the strength of the block and mortar being far lower than the pressure of the explosion product, a circular explosion pit centered on the explosive action area is formed on the explosion facing surface of the masonry wall under the action of a strong compressive load. Simultaneously, several radial cracks appear outside the explosion pit, and the cracks expand along the contact surface of the block and mortar, as shown in Figure 2a. The explosion pit on the blasting front face expands gradually with the propagation of the stress wave in the masonry wall. As shown in Figure 2b, the blasting back surface of the masonry wall begins to be damaged at 400 μs and forms an approximate elliptical through the hole. The long axis of the through hole was along the vertical direction, the short axis was along the horizontal direction, and cracks distributed along with the interface between block and mortar also appeared outside the through hole of the blasting back surface. Figure 2c–e shows the expansion process of the through hole. With the expansion of the through hole, the surface cracks of the masonry wall also gradually expand outward. The expansion process is completed at 800 μs, and the failure state of masonry wall will not change.

### 2.3. Damage of the Wall under Different Doses of Explosives

**Figure 3:**
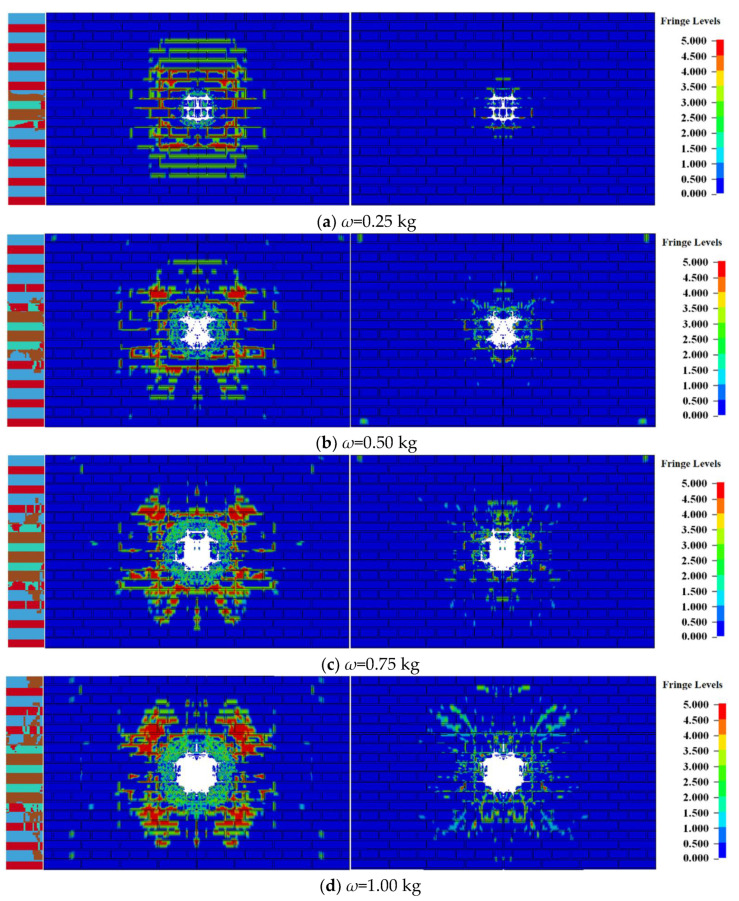
Damage of the 240 mm wall with different doses of explosives.

Figure 3 shows the damage of the 240 mm wall at *t* = 1000 μs under different amounts of TNT (0.25–1.00 kg) of explosive contact.

Figure 3a–d shows the damage morphology of the 240 mm masonry walls under contact explosions with different doses of explosives of 0.25 kg, 0.5 kg, 0.75 kg, and 1.00 kg. With the mass of charge increase, the area of the explosion pit, through the hole and crack zone on the surface of the masonry wall, gradually increases, and the damage of the masonry wall intensifies. From the shape of the through hole, when the mass of charge is small, the hole shape is oval, and the long axis of the ellipse is along the vertical direction. When the mass of charge increases, the hole shape gradually becomes round. This is because the strength of the block is greater than that of the mortar. When the load is small, the shape of the through hole is greatly affected by the uneven strength of the masonry wall, and its boundary is distributed at the interface between the block and the mortar. When the load strength is much greater than the masonry strength, the impact of the uneven strength of the masonry wall on the shape of the through hole decreases, and the hole shape gradually approaches being circular. The distribution of cracks also shows a similar law. When the mass of charge is small, the cracks on the surface of the masonry wall are distributed along with the interface between the block and mortar, but with the mass increase, the crack distribution is no longer affected by the boundary. The main crack shape is similar to the crack distribution of uniform medium, but the cracks along the boundary between the block and mortar can still be observed at the crack tip. 

**Figure 4:**
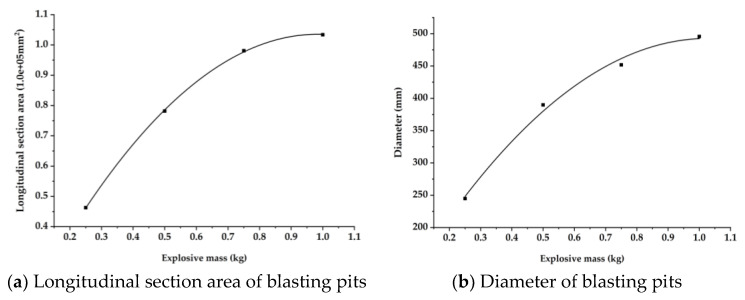
Statistics of the longitudinal section size of the 240 mm wall under different doses of contact explosion.

Figure 4 shows the damage sizes of the blasting pits of the masonry wall under different doses of contact explosion. As shown in Figure 4a, the volume of the blasting pit basically increased linearly when the explosive charge increased from 0.25 kg to 1 kg. When the explosive charge was more than 0.5 kg, the slope of the line decreased significantly, increased slowly, and tended to be horizontal although the volume also increased. In Figure 4b, the diameter d of the blasting pit basically increased linearly when the explosive charge increased from 0.25 kg to 0.5 kg. When the explosive charge was more than 0.5 kg, the diameter d also increased slowly, approaching the horizontal line.

In the numerical simulation of the 240 mm wall, when the explosive charge is less than 0.5 kg, the wall deformation mainly appeared in the form of blasting pits and longitudinal cracks. When the explosive charge was more than 0.5 kg, the dominant mode of energy release of the masonry wall changed when the charge was increasing; that is, when the central blasting pit of the wall expanded to a certain extent and the wall was penetrated, the front surface of the wall was covered with cracks, and the back surface was covered with annular cracks along with the mortar joints.

### 2.4. Simulation Result Analysis

According to the numerical simulation results, the following conclusions are obtained:

(1) Under contact explosion, when the mass of TNT was less than 0.5 kg, the damage form of the masonry wall was mainly reflected in the formation of the central blasting pit, penetrating hole, and cross cracks in horizontal and vertical directions. When the charge was greater than the critical value, the damage of the explosive to masonry wall gradually spread to the surroundings, especially the surrounding mortar joints; the spalling and collapse of the blast back surface also increased significantly, and the wall would eventually collapse.

(2) Under the contact explosion, the increase in the size of the blasting pit of the 240 mm wall gradually stopped after the dose was greater than 0.5 kg. Thereafter, the energy of the explosive load was released by splashing wall fragments, deflecting, and movement of the wall. The critical dose penetrating the 240 mm wall was 0.5 kg, which is the critical amount for the transformation of the dominant mode of energy release of the masonry wall.

## 3. Numerical Simulation of Blast-Resistant Performance of Spraying Polyurea to Reinforce 240 mm Wall under Contact Explosion

This section studies the influence of the polyurea reinforced layer on the blasting back surface with different thickness on the damage of the 240 mm wall under the contact explosion of a 0.5 kg TNT explosive. Four schemes were proposed, as shown in Table 4.

### 3.1. Damage of Sprayed Polyurea Wall at Different Times

**Figure 5:**
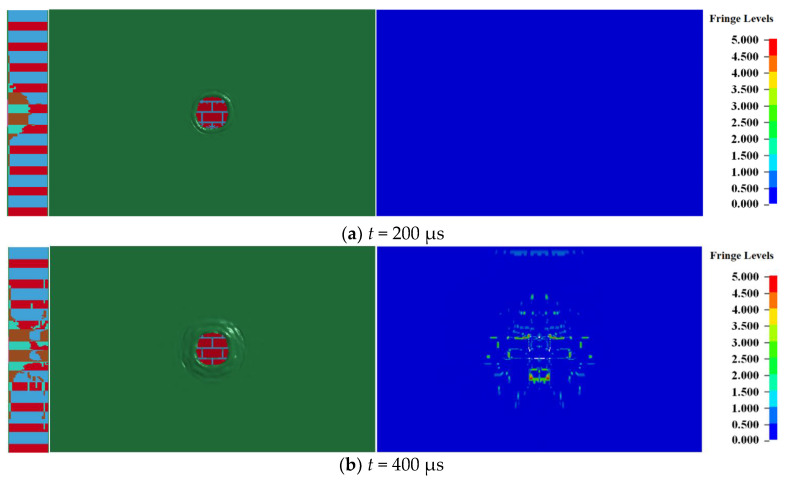

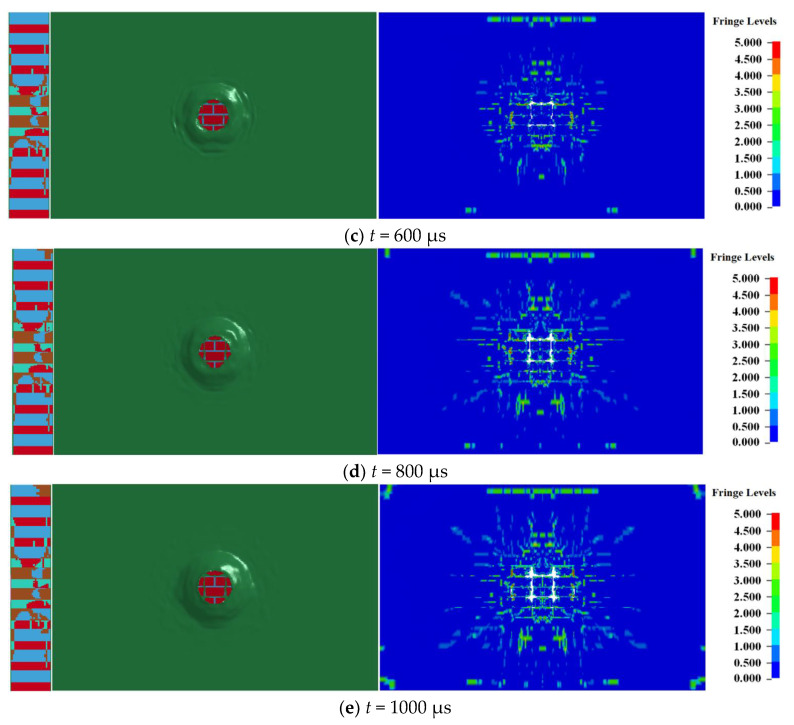
Destruction of the polyurea-reinforced 240 mm wall at different times.

When the amount of TNT was 0.5 kg under scheme 2, the damage of the 240 mm wall at different times is shown in Figure 5.

At the initial moment of contact explosion, the high-temperature and high-pressure detonation products are loaded onto the detonation facing surface of the masonry wall, so that the polyurea layer is eroded and a circular penetration pit is formed. The diameter of the penetration pit gradually expands with time, as shown in Figure 5a. Then, the stress wave is introduced into the masonry wall, and the blocks and mortar begin to break. However, due to the constraints of the polyurea layer, the formed fragments cannot fly out. The bulge around the polyurea layer in Figure 5b is formed by interacting with the fragments with an outward movement trend. When the strength of the stress wave is not enough to crush blocks and mortar, cracks will form along the wave propagation direction. The fragments formed on the back explosion surface are also completely wrapped by the polyurea layer. The damaged area of the masonry wall will not change at 800 μs. Compared with Figure 2, after the polyurea layer is added on both sides of the masonry wall, although the masonry wall will still be damaged and form a large number of fragments when the explosion occurs, most of the fragments are wrapped inside the polyurea layer and will not fly out, causing secondary damage to personnel or equipment. In addition, the deformation of the polyurea layer will absorb part of the explosion energy and reduce the damage on both sides of the masonry wall. The diameter of the through hole of the masonry wall in Figure 5e is 30.4% lower than that in Figure 2e. Most of the cracks on the blasting surface expand along the boundary of the masonry and mortar, and there are no outward radial cracks, which proves that the strength of the stress wave acting on the blasting surface of the masonry wall is significantly reduced.

### 3.2. Damage of Wall Sprayed with Polyurea under Different Working Conditions

**Figure 6:**
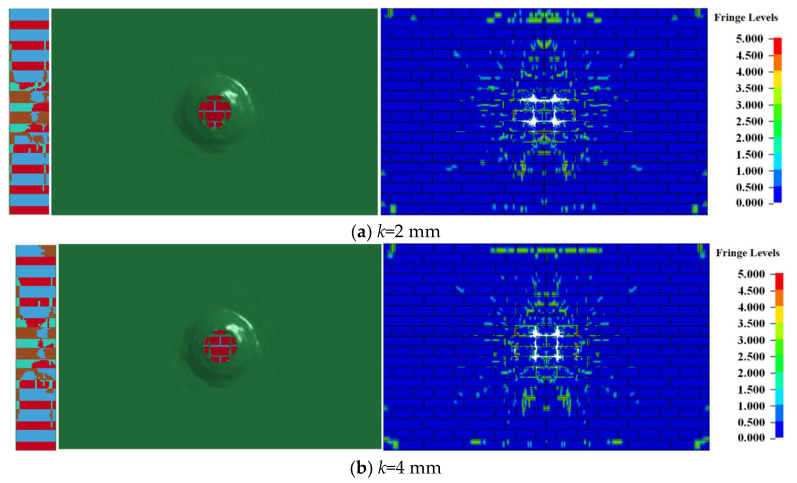

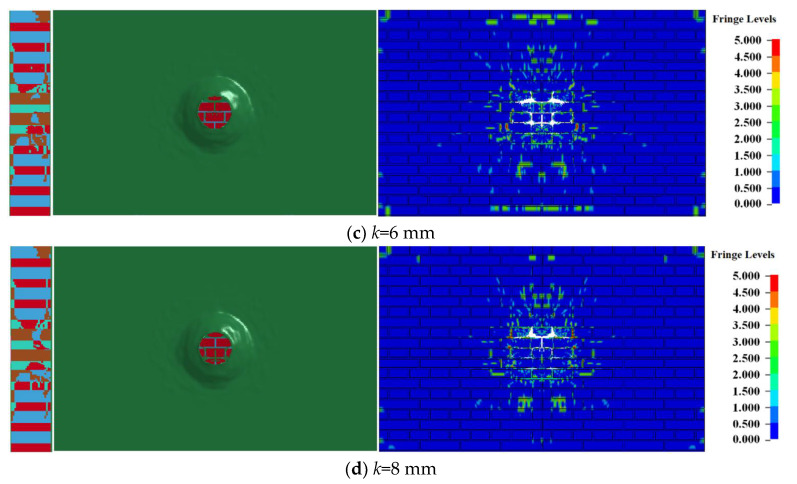
Damage of the reinforced 240 mm wall under contact explosion.

Figure 6 shows the damage of the reinforced 240 mm wall under contact explosion at *t* = 1000 μs. The labels (a–d) are the failure conditions of working conditions 1–4, respectively, and the polyurea reinforcement layers on the right sides were omitted in Figure 6 to better observe the damage of the reinforced masonry wall under contact explosion. Only shown are the damage of bricks and mortar under the four working conditions.

Comparing the damage of masonry wall under four working conditions, it is found that increasing the thickness of the polyurea layer on the back blasting surface, the diameter of the through hole and the range of the crack area on the back blasting surface gradually decrease. On the blasting face, due to the same amount of explosive and the thickness of the polyurea layer, the perforation diameter of the polyurea layer on the blasting face and the size of the crushing area of an internal masonry wall is 19.8 mm and 48.1 mm. The results of the front damage area and side section damage area of the masonry wall is shown in Table 5. The damage parameter values obtained under the four working conditions are very close. The above results show that only increasing the thickness of polyurea coating on the back blasting surface of masonry wall has little effect on the damage of the blast facing surface and side section of a masonry wall, but the damage of the back blasting surface will be significantly reduced. When the coating thickness is 8 mm, the damage is reduced by 54.5% compared with that when the coating thickness is 2 mm.

## 4. Test Verification of Spraying Polyurea to Reinforce 240 mm Wall under Contact Explosion

### 4.1. Test Plan and Results

The materials such as sprayed polyurea and reinforced masonry wall were the same as that in the previous section, and the masonry wall size was 2000 mm × 1200 mm × 240 mm [11]. The explosive used in the test was TNT cylindrical compressed explosive with a density of 1.63 g/cm^3^, a mass of 0.50 kg, and a size of Φ100 mm × 39 mm. The blast front surface of the reinforced 240 mm wall was sprayed with a 6 mm-thick polyurea reinforcement layer and the back surface was sprayed with a polyurea reinforcement layer of 4 mm and corresponded to working conditions 2 in the simulation.

**Figure 7:**
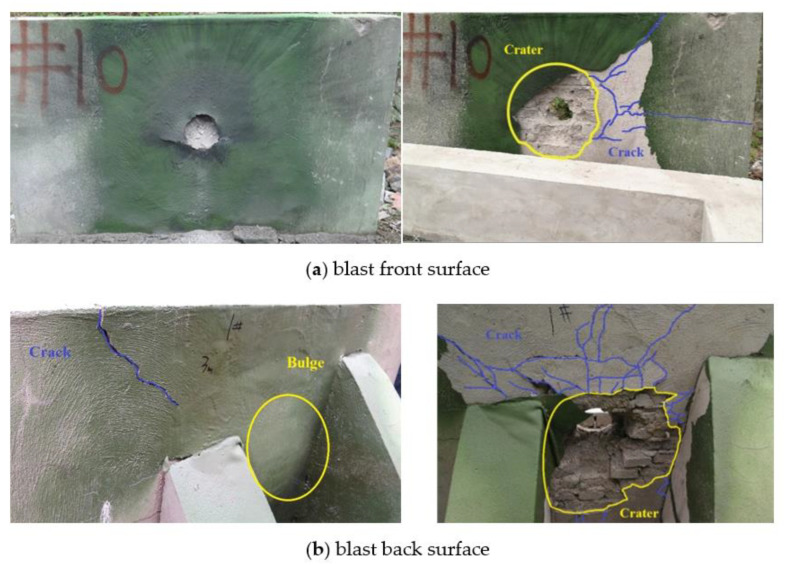
Damage to the reinforced 240 mm wall in the test.

The damage of the reinforced wall in the test is shown in Figure 7. The right side of Figure 7 shows the damage to the wall after removing the reinforcement layer. The center of the polyurea coating on the front surface of the reinforced wall produced a hole, which was slightly larger than the bottom surface of the explosive, with several short tears on both sides of the hole. The damaged masonry wall can be seen through the breach of the reinforcement layer, and some wall fragments, debris, and dust were “trapped” in the gap between the polyurea reinforcement layer and the masonry wall. Large blasting pits with shallow edges were formed in the center of the internal masonry wall. The part of the wall corresponding to the position of the explosive was penetrated. Many small spidery cracks and spalls developed along the mortar joints were produced around the blast front surface blasting pit. In addition, a large and penetrating crack was created above the blasting pit (the right of Figure 7a and the left of Figure 7b). As shown in the left side of Figure 7b, the central part of the blast back surface of the 240 mm wall was greatly uplifted, and a large number of damaged elements such as wall fragments were wrapped inside. The blast back surface of the internal masonry wall produced a large number of cracks that came from the through-hole, but most of the large cracks failed to tear the polyurea elastomer reinforcement layer of the blast back surface, as shown in Figure 7b.

### 4.2. Comparative Analysis

The numerical simulation of the reinforced 240 mm wall was compared with the unreinforced 240 mm wall under 0.5 kg TNT contact explosion; the damage of the masonry wall reinforced by polyurea elastomer under the working conditions 2–4 of the simulation was improved under the same dosage. Although the cracks inside the wall surface are increased and the area of the blasting pit was enlarged, the depth of the blasting pit and the area of the through-hole were significantly reduced, which effectively improves the support of the wall and makes it difficult to deflect and collapse. The area of the through hole in the center of the reinforced wall in working condition 1 was larger than that of the bare wall, but the polyurea elastomer reinforcement layer on the blast back surface can well wrap the damage element, absorb shock waves and provide stronger support in this case; the integrity of the wall in working condition 1 will be better than that of the bare wall.

From the perspective of the phenomenon, the numerical simulation showed the process of large blasting pits forming, the through hole creating in the center of the blast pit. The small cracks at the edge of the pits developed along the mortar joints, and the coarse cracks gradually extended and even penetrated along the mortar joints in the horizontal and vertical directions when the reinforced masonry wall was under the contact explosion load. The damage state was consistent with the test, indicating that the wall model sprayed with the polyurea reinforcement layer and polyurea material model can reflect the actual situation of the dynamic response of the reinforced masonry wall under contact explosion load.

Table 6 compares the damage parameters of the 240 mm bare wall and sprayed polyurea masonry wall under 0.5 kg charge. From the damage results, the damage of the blasting face of masonry wall strengthened by spraying polyurea is larger than that without strengthening, while the damage of the longitudinal section and back blasting face wall is significantly reduced. This is because after spraying polyurea on the surface of the masonry wall, the ductility of the wall increases, and the area of the blasting face participating in deformation and energy consumption also increases, so the damage of the blasting face of the wall intensifies. When the thickness of the polyurea layer is increased, the damage changes of the blasting face and longitudinal section of the wall are not obvious, but the damage area of the back blasting face decreases. Compared with the unreinforced masonry wall, after spraying polyurea, the maximum damage increase of the blasting face is 53.3%, the maximum damage decrease of the longitudinal section is 7.2%, and the damage decrease of the blasting face behind the 8 mm coating of polyurea is 55.6%. The above results show that spraying polyurea on the surface of the masonry wall can significantly reduce the damage of the back blasting surface of the masonry wall, and the damaged bricks of the blasting surface will be wrapped by the polyuria of the wall surface. Therefore, the masonry wall can maintain the structural integrity, which is very beneficial to the anti-explosion of the structure and the protection of personnel on the back explosion surface of the structure. At the same time, Table 5 compares the damage parameters of masonry walls obtained from numerical simulation and verification test under working condition 2. It can be concluded that the relative error of damage of the wall facing explosion surface is 5.8% and that of the back explosion surface is 2.5%. The results of the numerical simulation and verification test are in good agreement, which proves that the analysis of wall damage parameters is effective.

## 5. Conclusions

In this paper, the masonry wall with polyurea coating method under unidirectional support and different strength contact explosion load was numerically simulated, and some working conditions were verified by experiments. The failure characteristics and damage mechanism of reinforced masonry walls under contact explosion were analyzed, and the following conclusions were obtained:

(1) Simultaneously spraying polyurea elastomer on the front and back surfaces of the masonry wall can improve the support and integrity of the wall effectively, thereby resisting the damage caused by the contact explosion load.
(2) When the reinforcement form of the reinforcement layer on the blasting front surface remained unchanged, increasing the thickness of the polyurea layer on the blasting back surface can achieve a corresponding substantial decrease in the damage of the blasting back surface, while it has little change in the damage on the blasting front face and longitudinal section.
(3) The numerical simulation shows that under the contact explosion load, the reinforced masonry wall formed a breach and a central blasting pit on the reinforcement layer of the blast front surface. Small cracks were distributed around the blasting pits, and coarse cracks gradually extended along the mortar joints in the horizontal and vertical directions until penetration. The numerical simulation results were in good agreement with the experimental results.

## Figures and Tables

**Table 1:** Material parameters of explosives (g-cm-μs).

MID	RO	*D*	*P*_CJ_	BETA	*K*	*G*	SIGY
——	1.63	0.693	0.27	0.00	0.00	0.00	0.00

**Table 2:** Parameters of explosive (EOS_JWL) (g-cm-μs).

EOSID	*A*	*B*	*R*_1_	*R*_2_	OMEG	*E*_0_	*V*_0_
——	3.71	0.0743	4.15	0.95	0.3	0.07	1.00

**Table 3:** Material parameters of blocks and mortar.

Material	Density (kg/m^3^)	Elastic Modulus (MPa)	Poisson’s Ratio	Tensile Strength (MPa)	Shear Strength (MPa)	Compressive Strength (MPa)	Fracture Toughness (N/m)	Shear Transfer Coefficient
Block	1150	380	0.15	1.00	0.50	17.6	120	0.03
Mortar	2100	4644	0.25	1.76	0.90	9.0	140	0.03

**Table 4:** Thickness size of polyurea reinforced layer on 240 mm wall model.

Serial Number	Thickness of Front Surface/mm	Thickness of Back Surface/mm
1	6	2
2	6	4
3	6	6
4	6	8

**Table 5:** Size statistics of the destruction of the reinforced 240 mm wall under contact explosion.

Serial Number	Thickness of Front Surface (mm)	Thickness of Back Surface (mm)	Front Area of Blasting Pit (mm^2^)	Longitudinal Section of Blasting Pit (mm^2^)	Back Area of Blasting Pit (mm^2^)
1	6	2	183,352.6	73,464.95	58,402.53
2	6	4	182,857.79	76,436.16	50,759.39
3	6	6	181,742.59	77,193.85	43,695.06
4	6	8	182,442.40	76,758.36	26,602.17

**Table 6:** Comparison of parameters for the destruction of the reinforced 240 mm wall under contact explosion.

Serial Number	Polyurea Thickness on the Front Surface (mm)	Polyurea Thickness on the Back Surface (mm)	Positive Area of Blasting Pit (mm^2^)	Longitudinal Section of Blasting Pit (mm^2^)	Back Area of Blasting Pit (mm^2^)
Unreinforced	0	0	119,289.18	79,148.96	59,907.76
Working condition 1	6	2	183,352.6	73,464.95	58,402.53
Working condition 2 /test	6	4	182,857.79/ 194,042.40	76,436.16/—	50,759.39/ 52,036.41
Working condition 3	6	6	181,742.59	77,193.85	43,695.06
Working condition 4	6	8	182,442.40	76,758.36	26,602.17

## Data Availability

Data are contained within the article.
